# Endothelial estrogen receptor alpha (ESR1) regulates cerebral cavernous malformation pathogenesis via MEKK3–KLF signaling pathway

**DOI:** 10.1177/0271678X261468891

**Published:** 2026-07-06

**Authors:** Hamidreza Sadegh, Liwenyu Chen, Suyun Yu, Sobia Idrees, Matthew Foley, Keshav Raj Paudel, Jia Li, Yang Zhao, Jaesung P Choi

**Affiliations:** 1 Faculty of Science, School of Life Sciences, Centre for Inflammation, Centenary Institute and University of Technology Sydney (UTS), Sydney NSW, Australia; 2 School of Medicine, Nanjing University of Chinese Medicine, Nanjing, China; 3 Sydney Microscopy & Microanalysis, The University of Sydney, Camperdown, NSW, Australia; 4 NICM Health Research Institute, Western Sydney University, Westmead, NSW, Australia; 5 Curtin Medical School, Curtin Medical Research Institute, Curtin University, Bentley, WA, Australia; 6 Jiangsu Key Laboratory for Pharmacology and Safety Evaluation of Chinese Materia Medica, Nanjing University of Chinese Medicine, Nanjing, China; 7 School of Biomedical Sciences, UNSW Sydney, Kensington, NSW, Australia

**Keywords:** Cerebral cavernous malformation, KRIT1, ESR1, estrogen, stroke

## Abstract

Cerebral cavernous malformations (CCMs) are common brain hemangioma that can occur sporadically or be inherited. CCM is one of the major causes of hemorrhagic stroke and neurological deficits in children. There are no pharmacological treatments for CCM. Clinical observations suggest that estrogen may have important roles in CCM, however, it has not been investigated. Hence, we investigated the role of estrogen and its nuclear receptors estrogen receptor-α (*Esr1*) in experimental CCM. To determine the role of endothelial ESR1 in CCM, we crossed homozygous endothelial *Esr1* (*Esr1^fl/fl^*) mice into *Ccm1^iECKO^* mice. Micro-computed tomography (micro-CT) imaging was used to analyze CCM burden. To determine the therapeutic potential of estrogen, we treated *Ccm1^iECKO^* mice with *estradiol (E2, a* clinically approved estrogen). Gene and protein expressions were assessed in human umbilical vein endothelial cells (HUVECs). Homozygous deletion of endothelial *Esr1* in *Ccm1^iECKO^* mice significantly increased CCM lesion volume compared to littermate controls. KLF2/4 and downstream expressions in HUVECs were further increased by ESR1 depletion. This correlated with increased lesion burden in *Ccm1^iECKO^Esr1^fl/fl^* mice. Furthermore, we demonstrated E2 treatment in *Ccm1^iECKO^* mice prevented CCM pathogenesis by normalizing KLF2/4 and downstream expressions. Our study demonstrates ESR1 as a novel targeted therapeutic option for CCM.

## Introduction

Cerebral cavernous malformations (CCMs), also known as cavernous hemangioma, are relatively common brain vascular malformations with a prevalence of up to 0.5% of the population.^1,2^ At the ultrastructural level, CCMs manifest as clusters of thin-walled dilated vessels composed of abnormal cystic vascular channel lined by a monolayer of endothelial cells (EC) with impaired tight junctions^3,4^ and disorganized endothelium.^5^ Although many remain dormant, CCM lesions can lead to intracranial hemorrhage causing stroke, severe headaches, seizures, and neurological deficits.^6^ Currently, no drug treatments are available to prevent or treat CCM disease, and high-risk neurosurgery remains the only option and even that only when lesions are surgically accessible. Hence, there is an urgent need for non-invasive and safe treatment options.

CCM is an inherited dominant disorder due to loss-of-function mutations in one of three genes: *CCM1* (aka *KRIT1*), *CCM2* or *CCM3* (aka *PDCD10*).^2,7,8^ *CCM* genes encode non-homologous cytoplasmic proteins forming a single signaling complex.^9–11^ Recent studies showed that this CCM protein complex suppresses the Mekk3–Klf2/4–Adamts5/Rho signaling pathway, which drives CCM pathogenesis.^12–15^ Genetic deletion of MEKK3–KLF–ADAMTS signaling prevents CCM lesion formation in mouse models.^5,12,15–17^

Several clinical studies from different groups reported the existence of sex differences in CCM pathogenesis and potential roles for female hormones (e.g. estrogen) in CCM disease.^18–24^ However, these observations remain controversial and have not been experimentally investigated in CCM disease. Hence, this study is aimed to determine the role of estrogen in experimental CCM pathogenesis and test the therapeutic potential of estradiol (E2), a clinically approved estrogen currently used as hormone therapy, as an effective treatment in experimental CCM.

## Methods

### Study design

This study was designed to define the role of estrogen in CCM disease and its therapeutic potential. Endothelial-specific *Ccm1* knockout (*Ccm1^iECKO^*) mice were crossed into *Esr1* conditional (*Esr1^fl/fl^*) mice to determine the role of Esr1 in CCM pathogenesis. Furthermore, *Ccm1^iECKO^* mice were pharmacologically treated with E2 to determine the therapeutic potential of estrogen in CCM pathogenesis. All studies used appropriate (Cre-negative) littermate controls. Mice were euthanized by carbon dioxide (CO_2_) inhalation. CCM disease burden was examined by micro-computed tomography (micro-CT) imaging to assess changes in lesion volume and number. To study molecular mechanisms, Ccm1 was knocked in primary human umbilical vein endothelial cells (HUVECs) using siRNA and quantitative polymerase chain reaction (qPCR) and Western blot were used to measure gene expression changes.

### Mouse models

*Cdh5-CreERT2* PAC transgenic (iECre), *Ccm1^fl/fl^*, and *Esr1^fl/fl^* mice were previously described.^13,25–27^ The Sydney Local Health District Animal Welfare Committee approved all animal ethics and protocols. All experiments were conducted under the guidelines/regulations of Centenary Institute and the University of Technology Sydney, Australia. Animals received humane care in accordance with the Australian Government Authorities on the *NSW Animal Research Act 1985* and *Australian code for the care and use of animals for scientific purposes* (eighth edition 2013) issued by the National Health and Medical Research Council, the Australian Research Council, the Commonwealth Scientific and Industrial Research Organization and Universities Australia (“the *Code*”). Animal experiments are reported in accordance with ARRIVE 2.0 guidelines.

### Neonatal CCM mouse model and E2 administration

*Ccm1^iECKO^* and *Ccm1^iECKO^Esr1^fl/fl^*, and littermate control mice were generated by crossing *Cdh5-CreERT2Ccm1^fl/fl^Esr1^fl/+^* mice with either *Ccm1^fl/fl^Esr1^fl/+^* or *Ccm1^fl/fl^Esr1^fl/fl^* mice. To induce gene deletion, 50 µl of 4-hydroxytamoxifen (4-HT, 0.5 mg/ml; Sigma–Aldrich) was administered intragastrically to neonatal pups at postnatal day 1 (P1). β-Estradiol (E2; Sigma–Aldrich) was dissolved in pure ethanol to a 50 mg/ml stock and stored at −80 °C for up to 4 weeks. On the day of injection (P6), stock E2 (50 mg/ml) was diluted in sesame oil (Sigma–Aldrich) to intragastrically deliver 50 µl of E2 (at 25, 50, or 100 μg/kg dose) to E2-treated groups. Sham-treated groups were vehicle injected (50 μl of sesame oil).

### Micro-CT scan and analysis

Samples were prepared for micro-CT imaging using methods as previously described.^16,28^ Scans were performed using a SkyScan 1272 micro-CT scanner with a beam operating at 65 kV and 153 µA. A 1 mm aluminum filter was used to minimize beam hardening. A pixel size of 10.4 µm was chosen to optimally fit the samples across their various dimensions into a single scan. The resulting projection images were reconstructed into a stack of cross-sections using NRecon (v2.2.06) with all relevant parameters kept constant for consistency in analysis. The image stacks were then analyzed using Avizo as previously described.^16,28^

### Cell culture and E2 administration

Primary human umbilical vein endothelial cells (HUVECs) were freshly isolated from umbilical vessels as previous described^29^ and approved by the Medical Ethical Committee of Jiangsu Province Hospital on Integration of Chinese and Western Medicine (permit and approval number: 2021-LWKY-003). The study was conducted in accordance with the ethical principles outlined in the Belmont Report, and written informed consent was obtained from all participants. siRNA-mediated gene knockdown (*Ccm1* and *Esr1*) was performed as previously described.^5^ Following transfection, HUVECs were grown to approximately 60% confluence in complete Medium 199 (cat. no. 10-060-CV; Corning). Cells were then treated with 17-β-estradiol (E2, cat. no. E8875; Merk) at concentrations of 1, 10, or 100 nM. After 24 h of E2 administration, total RNA was extracted using the FreeZol Reagent (cat. no. R711; Vazyme) according to the manufacturer’s protocol. RNA samples were stored at −80 °C or immediately processed for downstream applications including RT-qPCR analysis.

### Gene expression analysis and Western blotting in cultured endothelial cells

PCR reactions were set up in triplicate and data shown are from ⩾3 independent experiments. The primers of genes are listed in Supplemental Table 1. Western blots were performed using a standard protocol with GAPDH (cat. no. AP0063; Bioworld) and KLF2 (cat. no. ab194486; Abcam) antibodies.

### Immunofluorescence staining

Mouse brains were fixed with 4% paraformaldehyde for 15 min at room temperature and subsequently rinsed three times with phosphate-buffered saline (PBS). Following fixation, samples were permeabilized with 0.5% Triton X-100 in PBS for 10 min and then blocked with 5% bovine serum albumin (BSA) in PBS for 1 h at room temperature to reduce nonspecific binding. The sections were incubated overnight at 4 °C with primary antibodies against CD31 (cat. no. 557355; BD) and ESR1 (cat. no. 20698-1-AP; Invitrogen) diluted in blocking buffer at optimized concentrations (e.g. 1:200 for CD31, 1:150 for ESR1). After three washes with PBS, samples were incubated with appropriate fluorophore-conjugated secondary antibodies Alexa Fluor™ 488 (cat. no. A11006; Invitrogen) or Alexa Fluor™ 594 (cat. no. A11012; Invitrogen) for 1 h at room temperature in the dark. Finally, nuclei were counterstained with DAPI for 5 min. After final washing, stained samples were mounted with antifade medium and imaged using a THUNDER Imager (Leica THUNDER Imaging Systems, Heidelberg, Germany).

### CCM patient RNA-seq data analysis

The RNA-seq raw data of 10 CCM samples from patients with brain stem CCM and four control samples from subjects with epilepsy were downloaded from GSE137596^30^ and levels of sex hormone receptors determined.

### Western blot

Cells transfected with the indicated siRNAs (si-CCM1 and/or si-ESR1) were lysed in RIPA buffer supplemented with protease and phosphatase inhibitor cocktail (Beyotime, China) for 30 min on ice. Lysates were centrifuged at 12,000*g* for 15 min at 4 °C, and the supernatants were collected. Protein concentrations were determined using a BCA Protein Assay Kit according to the manufacturer’s instructions. Equal amounts of protein (20–30 μg per lane) were separated by 10%–12% SDS–polyacrylamide gel electrophoresis (SDS–PAGE) and subsequently transferred onto polyvinylidene fluoride (PVDF) membranes (Millipore, USA) at 100 V for 90 min at 4 °C. Membranes were blocked with 5% non-fat dry milk in Tris-buffered saline containing 0.1% Tween-20 (TBST) for 1 h at room temperature to minimize nonspecific binding. The membranes were then incubated overnight at 4 °C with the following primary antibodies diluted in blocking buffer at optimized concentrations: anti-KLF2 (cat. no. ab236507; Abcam; 1:1000), anti-KLF4 (cat. no. 3674S; Cell Signaling Technology; 1:1000), anti-ADAMTS4 (cat. no. PA1-1749A; Invitrogen; 1:500), and anti-phospho-MLC2 (p-MLC2; cat. no. 3674S; Cell Signaling Technology; 1:1000). GAPDH was used as a loading control. After three washes with TBST (10 min each), membranes were incubated with the appropriate horseradish peroxidase (HRP)-conjugated secondary antibodies for 1 h at room temperature. Protein bands were visualized using an enhanced chemiluminescence (ECL) detection reagent (Thermo Fisher Scientific) and imaged with a chemiluminescence imaging system (Bio-Rad, USA). Band intensities were quantified using ImageJ software (NIH, USA) and normalized to the corresponding GAPDH signal.

### Cell viability assay

Cell viability was assessed using the Cell Counting Kit-8 (CCK-8) assay (Dojindo Molecular Technologies, Japan) according to the manufacturer’s instructions. Briefly, cells were seeded into 96-well plates at a density of 5000 cells per well and allowed to adhere overnight at 37 °C in a humidified atmosphere containing 5% CO_2_. Cells were subsequently transfected with the indicated siRNAs (si-CCM1 and/or si-ESR1) using Lipomaster 3000 transfection reagent (cat. no. TL301; Vazyme Biotech, Nanjing, China) following the manufacturer’s protocol. At 24 h post-transfection, 10 μl of CCK-8 reagent was added to each well containing 100 μl of culture medium, and the plates were incubated at 37 °C for 1 h in the dark. Absorbance was measured at 450 nm using a microplate reader (BioTek, USA). Relative cell viability was calculated by normalizing the absorbance of each experimental group to that of the negative control group (si-CCM1: −; si-ESR1: −), which was set as 100%. Statistical significance was determined by one-way ANOVA followed by Tukey’s post hoc test, with *p* < 0.05 considered statistically significant (ns: not significant; *p < 0.05).

### Transendothelial electrical resistance (TEER) measurement

Endothelial barrier integrity was evaluated by measuring transendothelial electrical resistance (TEER) using Millicell ERS-2 Electrical Resistance System (MilliporeSigma, Burlington, MA, USA). Briefly, endothelial cells were seeded onto 0.4 μm pore size polyethylene terephthalate (PET) Transwell inserts (Corning, USA) placed in 24-well plates at a density of 1.0 × 10^5^ cells per insert and cultured at 37 °C in a humidified atmosphere containing 5% CO_2_ until a confluent monolayer was formed. Cells were subsequently transfected with the indicated siRNAs (si-CCM1 and/or si-ESR1) using Lipomaster 3000 transfection reagent (cat. no. TL301; Vazyme Biotech, Nanjing, China) following the manufacturer’s protocol. TEER values were recorded at the designated time point post-transfection by placing the electrode vertically and perpendicular to the Transwell membrane, with the shorter prong submerged in the apical compartment and the longer prong in the basolateral compartment. The resistance of cell-free Transwell inserts containing culture medium alone was measured in parallel and subtracted as background. Final TEER values were expressed in Ω cm^2^ by multiplying the net resistance (Ω) by the effective membrane surface area (cm^2^). Statistical significance was determined by one-way ANOVA followed by Tukey’s post hoc test, with *p* < 0.01 considered statistically significant (**p < 0.01).

### Statistical analysis

All statistical analyses was performed in GraphPad Prism statistical software (version 9.5.1). No data points were excluded as outliers. All measurements were performed by investigators blinded to treatment groups. Data are presented as mean ± SD. Sample sizes (*n*) and statistical tests are given in the figure legends. Statistical significance was considered when *p* ⩽ 0.05.

## Results

### ESR1 is expressed in endothelial cells lining CCM lesions

**Figure 1.**
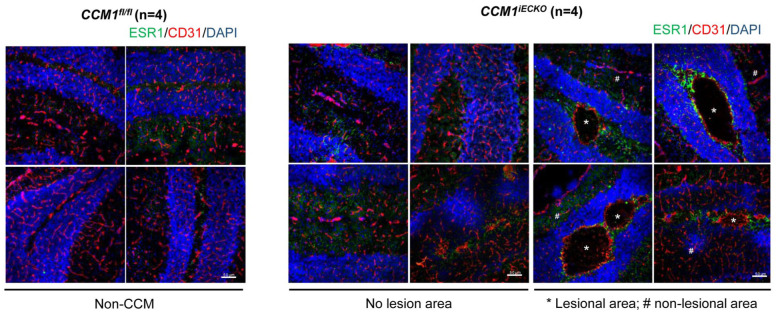
Immunofluorescence staining of ESR1 in mouse brains of *Ccm1^fl/fl^* and *Ccm1^iECKO^* mice. Representative immunofluorescence images of CD31 and ESR1 expressions in non-CCM (*Ccm1^fl/fl^*) and non-lesional and lesional areas from *Ccm1^iECKO^* mice. CD31 staining (red) denotes endothelial cells and ESR1 is labeled green.

Estrogen actions target tissues mainly by interacting with two nuclear receptors, estrogen receptor-α (*Esr1*) and -β (*Esr2*).^31^ A recent single-cell transcriptome study in mice^32^ demonstrated that both *Esr1* and *Esr2* are expressed in brain ECs, however, *Esr1* is more abundantly and widely expressed in different EC types in mouse brain than *Esr2* (Supplemental Figure I). Hence, we determined ESR1 expressions in brains of *Ccm1^fl/fl^* (non-CCM) and *Ccm1^iECKO^* (lesional and non-lesional areas) using immunofluorescence (Figure 1). Immunofluorescence showed that ESR1 is ubiquitously expressed in mouse brain tissues (non-CCM, non-lesional, lesional) and co-localized with CD31 (EC marker; Figure 1). Furthermore, to determine if *ESR1* and other sex hormone receptors such as *ESR2*, progesterone receptor (*PGR*) and androgen receptor (*AR*) gene expressions are altered in human CCM lesions, we searched and reanalyzed published transcriptomics data sets of human CCM samples which includes 10 CCM patients with brain stem CCM and four control subjects with temporal lobe epilepsy.^30^ Although the hormone receptors were expressed in resected brain samples from CCM patients, these expressions were not significantly different compared to control subjects (Supplemental Figure II).

### Endothelial *Esr1* deletion aggravates CCM progression

ESR1 is highly expressed in ECs of CCM lesions, hence, we aimed to determine if endothelial *Esr1* plays a role in the progression of experimental CCM disease. We deleted one or two alleles of *Esr1* (hereafter *Esr1^fl/+^* or *Esr1^fl/fl^*) in *Ccm1^iECKO^* mice.

**Figure 2.**
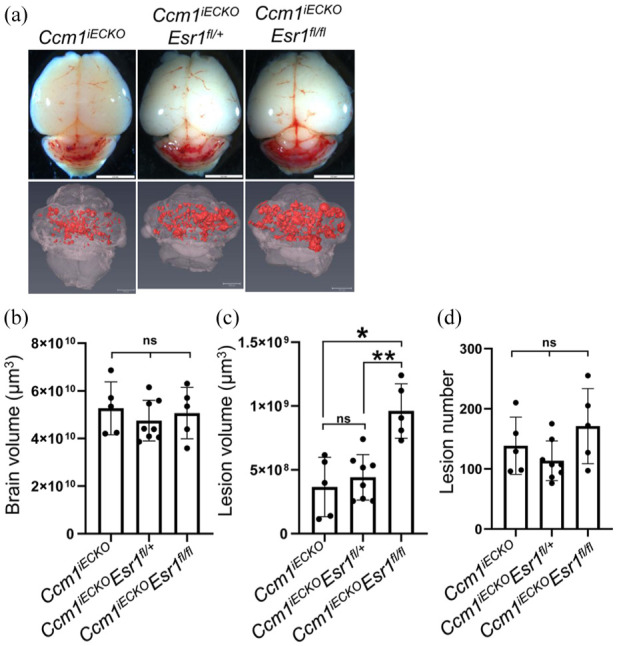
Endothelial *Esr1* deletion aggravated CCM lesion growth in *Ccm1^iECKO^* mice: (a) macroscopic (top) and micro-CT imaging (bottom) of brain CCM lesions in *Ccm1^iECKO^*, *Ccm1^iECKO^Esr1^fl/+^*, and *Ccm1^iECKO^Esr1^fl/fl^* at P12, (c–e) quantification of micro-CT analysis shows that endothelial *Esr1* deletion did not affect brain volume (b). Total lesion volume was significantly increased in *Ccm1^iECKO^Esr1^fl/fl^* brains compared to other groups (c), but total lesion number (d) was not changed across the groups. Error bars are shown as SD, statistical analyses were performed using one-way ANOVA, *n* ⩾ 5 each group. ns: not significant. *p < 0.001. **p < 0.005.

Vascular malformations were observed on the surface of the brains (Figure 2(a), top) of all three groups (*Ccm1^iECKO^*, *Ccm1^iECKO^Esr1^fl/+^*, and *Ccm1^iECKO^Esr1^fl/fl^*) at P12 and analyzed by micro-CT (Figure 2(a), bottom). Total brain volume in all groups was not changed (Figure 2(b)). *Ccm1^iECKO^Esr1^fl/fl^* mice had a two-fold increase in total lesion volume compared to *Ccm1^iECKO^* and *Ccm1^iECKO^Esr1^fl/+^* mice (Figure 2(c)). There was no difference between *Ccm1^iECKO^* and *Ccm1^iECKO^Esr1^fl/+^* mice. Despite dramatic increase in lesion volume, total lesion number was similar in all three groups (Figure 2(d)). This suggests that the homozygous endothelial ESR1 deletion may only affect the growth of existing lesions rather than initiation of new lesions. Endothelial ESR1 deletion alone did not cause CCM lesion formation in mice (Supplemental Figure III).

### ESR1 deletion augmented MEKK3–KLF signaling in *CCM1*-deficient HUVECs

Previous studies have shown that loss-of-function mutation of *Ccm* genes upregulates *Mekk3–Klf2/4* signaling to drive CCM pathogenesis.^12,17^ To determine if increased volumes of CCM lesions in *Ccm1^iECKO^Esr1^fl/fl^* mice were due to the augmentation of the *Mekk3–Klf2/4* signaling pathway, we used siRNA-induced knockdown of *CCM1* and *ESR1* genes in HUVECs and assessed the gene expression of the downstream *Mekk3–Klf2/4* pathway.

**Figure 3.**
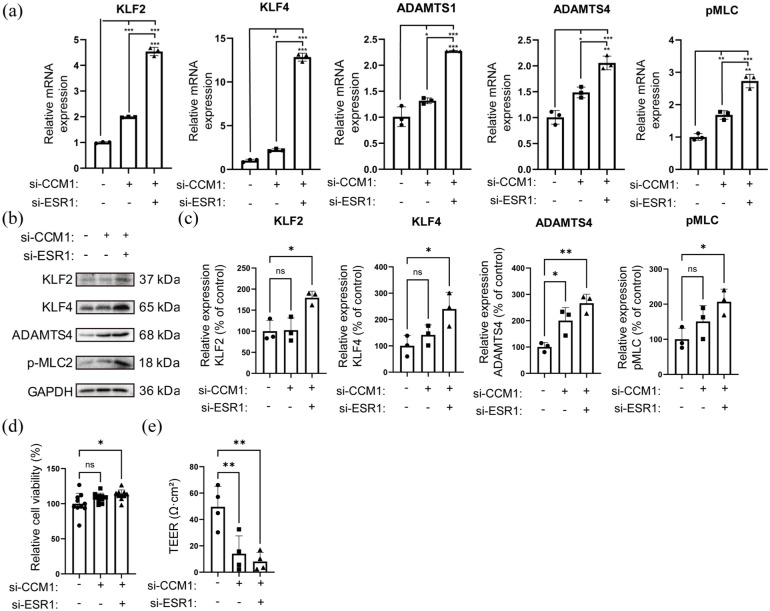
*ESR1* deletion augmented the expression of the MEKK3–KLF signaling pathway in *CCM1*-deficient HUVECs: (a) gene expression analysis of siRNA-knock down of *CCM1* (si-*CCM1*) and *ESR1* (si-*ESR1*) in HUVECs. CCM1 and ESR1 double knockdown further increased KLF2, KLF4, and the downstream pathway genes (ADAMTS1, ADAMTS4, and pMLC) compared to CCM1 knockdown alone, (b, c) representative Western blots (b) and quantification (c) showing that ESR1 knockdown increased KLF2, KLF4, ADAMTS4, and pMLC protein expressions, (d) cell viability assay showing CCM1 and ESR1 double knockdown increased relative cell viability (%) compared to control groups, and (e) TEER measurement showing si-CCM1 knockdown significantly decreased TEER measurement compared to control group. No difference between si-CCM1 and si-CCM1/ESR1 knockdown groups. Error bars are shown as SD and statistical analyses were performed using one-way ANOVA, *n* = 3 each group. ns: not significant. *p < 0.05. **p < 0.005. ***p < 0.0001.

As previously shown in our studies,^5,33,34^ si-*CCM1* treatment alone increased the mRNA expression of *KLF2*, *KLF4*, and their downstream target genes (*ADAMTS1*, *ADAMTS4*, and *pMLC*; Figure 3(a)) compared to scrambled siRNA controls. Double siRNA knockdown (si-*CCM1/ESR1*) further augmented the upregulated MEKK3–KLF signaling and its downstream genes. Increased mRNA expressions of these genes were also confirmed at protein level using Western blots (Figure 3(b) and (c)). Representative Western blots (Figure 3(b)) and the quantification (Figure 3(c)) show KLF2, KLF4, ADAMTS4, and pMLC protein levels were significantly increased in si-*CCM1/ESR1* group compared to the control group. This provides strong evidence that endothelial ESR1 deletion aggravated CCM lesions by further increasing the MEKK3–KLF signaling pathway.

Moreover, we performed cell viability assay (Figure 3(d)) and transendothelial electrical resistance (TEER) measurements (Figure 3(e)) to determine the functional properties of the endothelial cells following CCM1 and ESR1 deletion. Si-*CCM1* treatment alone did not change relative cell viability but double siRNA knockdown (si-*CCM1/ESR1*) significantly increased relative cell viability compared to the control group (Figure 3(d)). For TEER, which measures cell integrity and permeability, si-*CCM1* treatment alone and si-*CCM1/ESR1* significantly decreased TEER compared to the control group (Figure 3(e)). TEER was not significantly different between si-*CCM1* and si-*CCM1/ESR1* (Figure 3(e)).

### *Estradiol* inhibits CCM lesion initiation and growth

**Figure 4.**
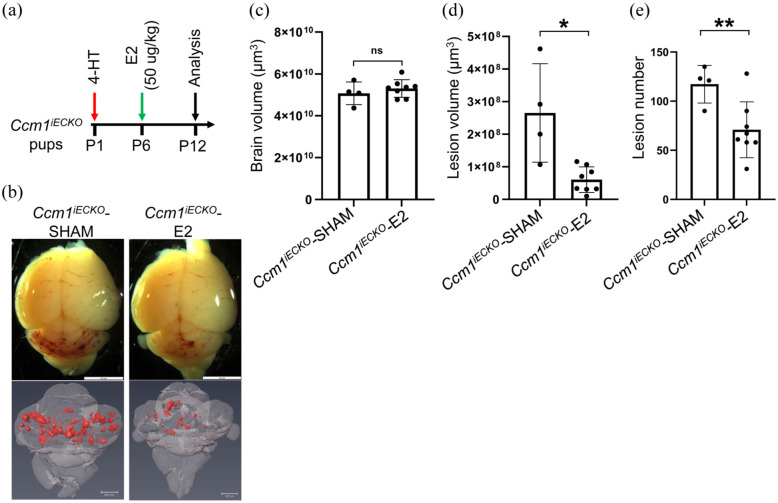
E2 inhibited CCM lesion formation and growth in *Ccm1^iECKO^* mice: (a) schematic of experimental design: gene deletion in neonatal pups (postnatal day 1) was induced with 4-HT. Mice were treated with E2 at P6 and brains were collected at P12 for analysis, (b) macroscopic (top) and micro-CT imaging (bottom) of CCM lesions in *Ccm1^iECKO^* with (left) or without (right) E2 treatment, (c–e) quantification of micro-CT analysis shows that E2 treatment in the *Ccm1^iECKO^* did not affect brain volume (c) but substantially reduced lesion volume (d) and number (e) compared to sham-treated littermate controls. Error bars shown as SD and significance were determined by *t*-test, *n* = 4 for sham groups and *n* = 8 for E2 treatment groups. E2: estradiol; 4-HT: 4-hydroxytamoxifen; ns: not significant. *p < 0.005. **p < 0.05.

Endothelial *Esr1* deletion aggravated the CCM disease burden in mice (Figure 2). Hence, we determined if *Esr1* can therapeutically targeted using clinically approved ESR1 agonist, E2.^35^ We delivered E2 (50 μg/kg) intragastrically to *Ccm1^iECKO^* mice at P6 and analyzed brains at P12 (Figure 4(a)).

In sham-treated *Ccm1^iECKO^* mice at P12, numerous vascular malformations were observed visualized by macroscopic and micro-CT imaging (Figure 4(b), left). Total brain volume in both groups was not changed (Figure 4(c)), suggesting that there was no gross brain abnormality upon E2 treatment. In contrast, and remarkably, a single dose of E2 at P6 in *Ccm1^iECKO^* mice substantially reduced disease burden with a 77% reduction in total lesion volume (Figure 4(d)) and 40% reduction in total lesion number (Figure 4(e)). The reduction of CCM lesion volume demonstrates that E2 is highly effective in preventing the growth of existing lesions (progression). The decrease in total lesion number indicates that E2 treatment prevented development of new lesion formation (initiation). Thus, E2 effectively ameliorates CCM lesion development in mice.

We also tested E2 at different doses (25 and 100 μg/kg), but CCM lesion burden was not affected at these doses (Supplemental Figure IV).

### Estradiol inhibited MEKK3–KLF signaling in CCM1-deficient HUVECs

To determine if E2 inhibition of CCM lesion formation and growth in *Ccm1^iECKO^* mice is through the regulation of *Mekk3–Klf2/4* signaling, we treated *si-CCM1* HUVECs with E2 and assessed the gene expression of the downstream *Mekk3–Klf2/4* pathway.

**Figure 5.**
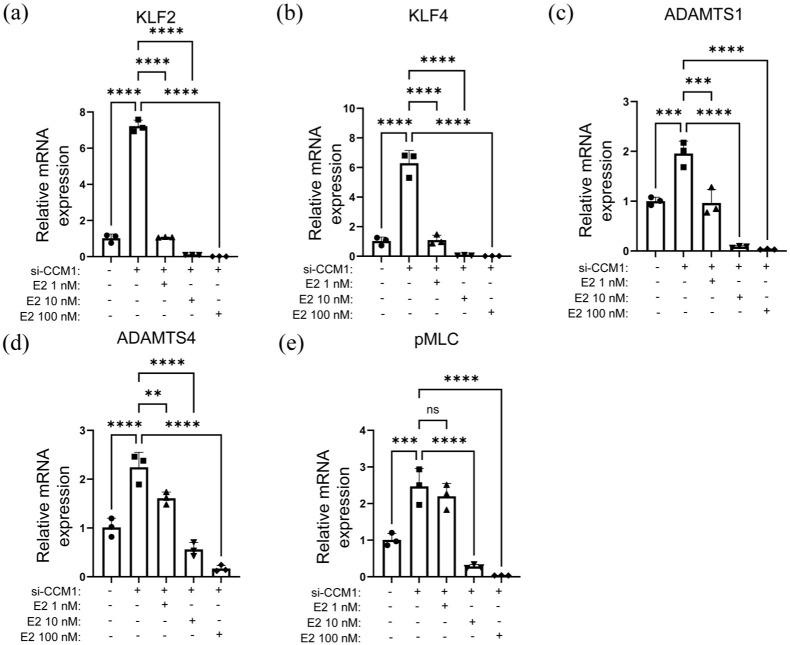
E2 treatment normalized MEKK3–KLF signaling pathway in CCM1-deficient HUVECs: (a–e) gene expression analysis of *si-CCM1* HUVECs treated with E2. E2 treatment in *si-CCM1* HUVECs normalized KLF2, KLF4, and the downstream pathway genes (ADAMTS1, ADAMTS4, and pMLC) compared to *si-CCM1* HUVEC controls. Error bars are shown as SD and statistical analyses were performed using one-way ANOVA, *n* = 3 each group. ns: not significant. **p < 0.05. ***p < 0.001. ****p < 0.0001.

As shown in Figure 3, *si-CCM1* treatment alone increased the mRNA expression of *KLF2, KLF4*, and their downstream target genes (*ADAMTS1*, *ADAMTS4*, and *pMLC*; Figure 5) compared to scrambled siRNA controls. Si-CCM1 HUVECs were treated with 1, 10, or 100 nM E2, which normalized the upregulated MEKK3–KLF signaling and its downstream genes in a dose-dependent manner (Figure 5). This suggests E2 inhibition of CCM lesion formation and growth in *Ccm1^iECKO^* mice is through normalized MEKK3–KLF signaling pathways in CCM pathogenesis.

## Discussion

Despite several clinical studies reporting sex differences and potential roles for sex hormones in patients,^18–24^ its role in CCM pathogenesis has not been investigated and remains controversial. Here, we used in vivo and in vitro CCM models to genetically and pharmacological manipulate estrogen actions to define its role in experimental CCM.

Estrogen actions target tissues by primarily interacting with *Esr1* and *Esr2.*^31^ In brain ECs, *Esr1* is more abundantly expressed than *Esr2* (Supplemental Figure I). Hence, we determined ESR1 expression in CCM lesions of *Ccm1^iECKO^* mice using immunofluorescence. Our results showed that ESR1 is ubiquitously expressed in CCM lesions including ECs in both the non-lesional and lesional areas (Figure 1). In humans, *ESR1* and other sex hormone receptors (*ESR2, PGR, AR*) were expressed in resected CCM patient brain samples, its expression level did not differ when compared to healthy controls (Supplemental Figure II).

Hence, we determined the functional role of endothelial *Esr1* in CCM pathogenesis by deleting endothelial *Esr1* in *Ccm1^iECKO^* mice. Our results demonstrated that homozygous endothelial ESR1 deletion aggravated CCM lesion burden (Figure 2). Micro-CT quantification revealed that the total lesion volume was significantly increased, whereas the total lesion number remained unchanged. Heterozygous endothelial ESR1 deletion did not affect CCM lesion burden. This suggests the complete loss of endothelial ESR1 increased CCM lesion burden by promoting growth of existing lesions rather than the formation of new lesions. Interestingly, homozygous endothelial ESR1 deletion alone did not induce the development of CCM lesions in the brain (Supplemental Figure III), further demonstrating that endothelial ESR1 does not play a role in CCM lesion development but only progression of the disease in the absence of CCM genes.

We further assessed how ESR1 regulates CCM pathogenesis. We knocked down ESR1 in CCM1-deficient HUVECs and measured components of the MEKK3–KLF signaling pathway, which is associated with driving CCM pathogenesis.^5,12,13,16,17^ In conjunction with aggravated CCM lesion burden upon endothelial ESR1 deletion in mice, *ESR1* knockdown in HUVECs augmented already upregulated MEKK3–KLF signaling and its downstream genes (Figure 3). Demonstrating that ESR1 acts upstream of the MEKK3–KLF signaling pathway and regulates its expression. However, we only confirmed MEKK3–KLF signaling pathway regulation using HUVECs. Hence, future studies in vivo models (e.g. mouse, human) are required to confirm the mechanism in CCM pathogenesis.

Interestingly, our findings also suggest that ESR1 and CCM1 in ECs could co-regulate each other as CCM1 knockdown significantly decreased ESR1 gene expression whereas ESR1 knockdown significantly increased CCM1 gene expression in HUVECs (Supplemental Figure V). This warrants further studies to elucidate the interactions of CCM1 and ESR1 genes.

As our study demonstrated that ESR1 via MEKK3–KLF signaling can regulate CCM pathogenesis, we tested the therapeutic potential of estrogen (ESR1 agonist) in CCM disease. Firstly, we tested different doses of E2 (25, 50, and 100 μg/kg) to determine the optimal dose for neonatal CCM mouse model. At 100 μg/kg dose of E2, 2/6 (33%) mice died after treatment suggesting its high toxicity (Supplemental Figure IV). Mice that received 25 or 50 μg/kg of E2 showed no signs of toxicity without any mortality. However, at 25 μg/kg of E2 did not affect CCM lesion burden. On the other hand, a single dose of E2 (50 μg/kg) at P6 dramatically reduced experimental CCM lesion burden (Figure 4). Micro-CT quantification revealed that both lesion volume and number were substantially reduced. This suggests that E2 treatment can prevent both the initiation of new lesions and the growth of existing lesions in neonatally induced experimental CCM. Using in vitro CCM model, we demonstrated the E2 inhibition of CCM lesion could be due to the normalization of MEKK3–KLF signaling pathway (Figure 5). Again, future studies in vivo models (e.g. mouse, human) are required to confirm the mechanism in CCM pathogenesis.

In support of our experimental findings, interestingly, in a cohort of familial “Common Hispanic Mutation” CCM patients, circulating blood aromatase levels inversely associated with disease burden.^36^ Aromatase in ECs converts androgens into estrogens thereby mediating the sex steroid influence in local modulation of blood-brain barrier permeability and cerebrovascular homeostasis.^37,38^ This suggests that increased circulating aromatase in CCM patients which increases the conversion of androgens into estrogens may contribute to reduced CCM disease burden. This supports our finding of E2 reducing CCM disease burden in neonatal mice.

Moreover, our study demonstrates a complex role of estrogen actions in CCM pathogenesis. Our pharmacological E2 treatment and genetic endothelial ESR1 deletion showed different effects on CCM lesion numbers. Pharmacological E2 treatment reduced both the lesion volume and number, regulating both the lesion progression and formation. On the other hand, genetic endothelial ESR1 deletion reduced lesion volume without changing lesion numbers, regulating only the lesion progression. In the pharmacological E2 treatment, estrogen is injected peritoneally, and it affects all the cell types (e.g. pericytes and other brain cell types), whereas genetic deletion of endothelial ESR1 only affects *Ccm1-null* ECs. This observation could be due to the cell-specific effect of estrogen where reduction in lesion number could be mediated by *Esr1* in cell types other than ECs. Furthermore, other estrogen signaling pathways such as ESR2 and non-genomics actions of estrogens (e.g. G protein-coupled estrogen receptor 1 or GPR30^39^) could play a role in estrogen actions in CCM pathogenesis, warranting further studies.

## Summary/conclusions

Our preclinical study demonstrated that genetic loss of endothelial ESR1 aggravated CCM lesion formation in *Ccm1*-deficient mice by further activating MEKK3–KLF signaling pathway. Moreover, E2 (ESR1 agonist) can effectively reduce CCM lesion burden in *Ccm1*-deficient mice. Collectively, our study suggests estrogen actions play a key role in experimental CCM and targeting ESR1 using clinically approved ESR1 agonist such as E2 could be a novel targeted therapy for CCM disease. Furthermore, our findings warrant further studies to elucidate the role of estrogen and other sex hormones (e.g. progesterone, androgens) in CCM pathogenesis. This is important and urgent as hormone replacement therapies (e.g. cancer therapy, endocrine therapy, contraceptives) are widely used in the clinic for many human diseases and may lead to undesired confounding effects on CCM patients.

## Supplemental Material

sj-docx-1-jcb-10.1177_0271678X261468891 – Supplemental material for Endothelial estrogen receptor alpha (ESR1) regulates cerebral cavernous malformation pathogenesis via MEKK3–KLF signaling pathway Supplemental material, sj-docx-1-jcb-10.1177_0271678X261468891 for Endothelial estrogen receptor alpha (ESR1) regulates cerebral cavernous malformation pathogenesis via MEKK3–KLF signaling pathway by Hamidreza Sadegh, Liwenyu Chen, Suyun Yu, Sobia Idrees, Matthew Foley, Keshav Raj Paudel, Jia Li, Yang Zhao and Jaesung P Choi in Journal of Cerebral Blood Flow & Metabolism
