# Supplementary material for: Endothelial estrogen receptor alpha (ESR1) regulates cerebral cavernous malformation pathogenesis via MEKK3–KLF signaling pathway
Source: J Cereb Blood Flow Metab. 2026 Jul 6:0271678X261468891. Online ahead of print. doi: 10.1177/0271678X261468891 (PMC13388494; doi:10.1177/0271678X261468891)
Supplement: sj-docx-1-jcb-10.1177_0271678X261468891 – Supplemental material for Endothelial estrogen receptor alpha (ESR1) regulates cerebral cavernous malformation pathogenesis via MEKK3–KLF signaling pathway [file sj-docx-1-jcb-10.1177_0271678X261468891.docx]

**Endothelial estrogen receptor alpha (ESR1) regulates cerebral cavernous malformation pathogenesis via MEKK3-KLF signalling pathway**

***Supplemental***

***Supplemental Table 1. Primers of genes used in this study.***

| **Gene** | **Forward primer** | **Reverse Primer** |
| --- | --- | --- |
| **GAPDH** | **GAGTCAACGGATTTGGTCGT** | **GATCTCGCTCCTGGAAGATG** |
| **CCM1** | **AATGGCAGAGAAGCATGAGCAGTG** | **ATTTCAGCATGTCCTCCTCCAGCA** |
| **ESR1** | **GAAAGGTGGGATACGAAAAGACC** | **GCTGTTCTTCTTAGAGCGTTTGA** |
| **KLF2** | **CTACACCAAGAGTTCGCATCTG** | **CCGTGTGCTTTCGGTAGTG** |
| **KLF4** | **GGCGGGCTGATGGGCAAGTT** | **TGCCGTCAGGGCTGCCTTTG** |
| **ADAMTS1** | **ACTGGAAGCATAAGAAAGAAGCG** | **AATTCTGCCATCGACTGGTCT** |
| **ADAMTS4** | **GAGGAGGAGATCGTGTTTCCA** | **CCAGCTCTAGTAGCAGCGTC** |
| **pMLC** | **TGCTGTGACCACTACGTGTTT** | **TGGCACTTCGTCCAGAATGTT** |

***Supplemental Figure I. Singe-cell transcriptomic analysis of mouse brain tissues.*** *Esr1* and *Esr2* is expressed in endothelial cells (EC) of adult mouse brain tissues. Data was sourced from <http://betsholtzlab.org/VascularSingleCells/database.html>. Abbreviations: PC - Pericytes; SMC - Smooth muscle cells; MG - Microglia; FB - Vascular fibroblast-like cells; OL - Oligodendrocytes; EC - Endothelial cells; AC - Astrocytes; v - venous; capil - capillary; a - arterial; aa - arteriolar; 1,2,3- subtypes.

**
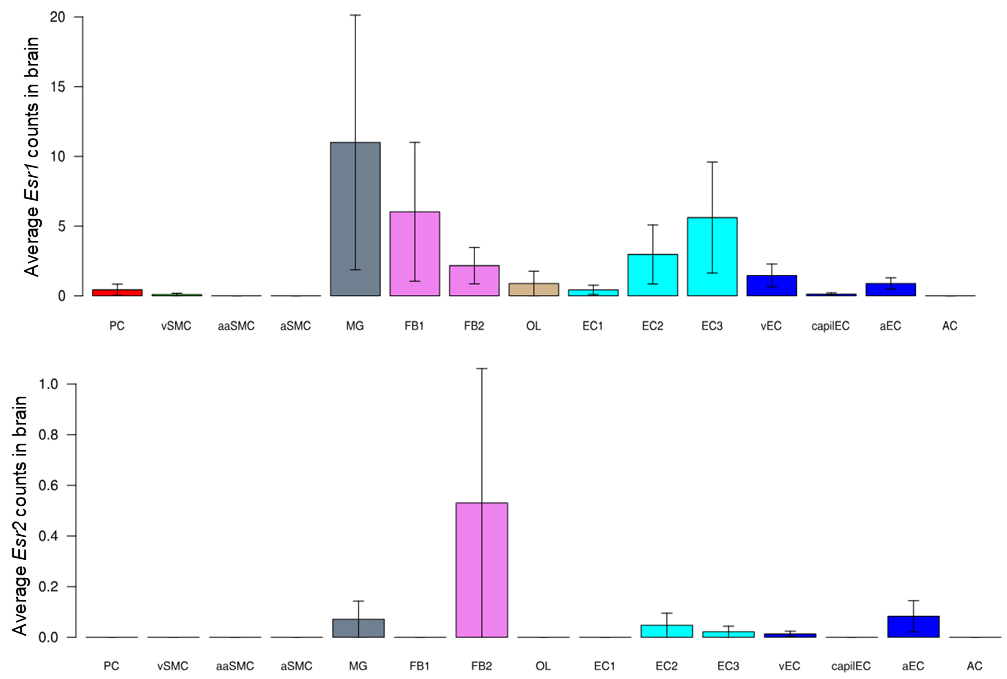
**

***Supplemental Figure II*. Transcriptome analysis of sex hormone receptors in human CCM tissues.** A-D) Relative mRNA expression of ESR1, ESR2, PGR, AR in CCM samples from patients with brain stem CCM compared to control samples from subjects with epilepsy. Error bars shown as SEM and significance was determined by t-test. * p<0.05, ns not significant. *n*=4 for control groups, *n*=10 for CCM patient groups.

**
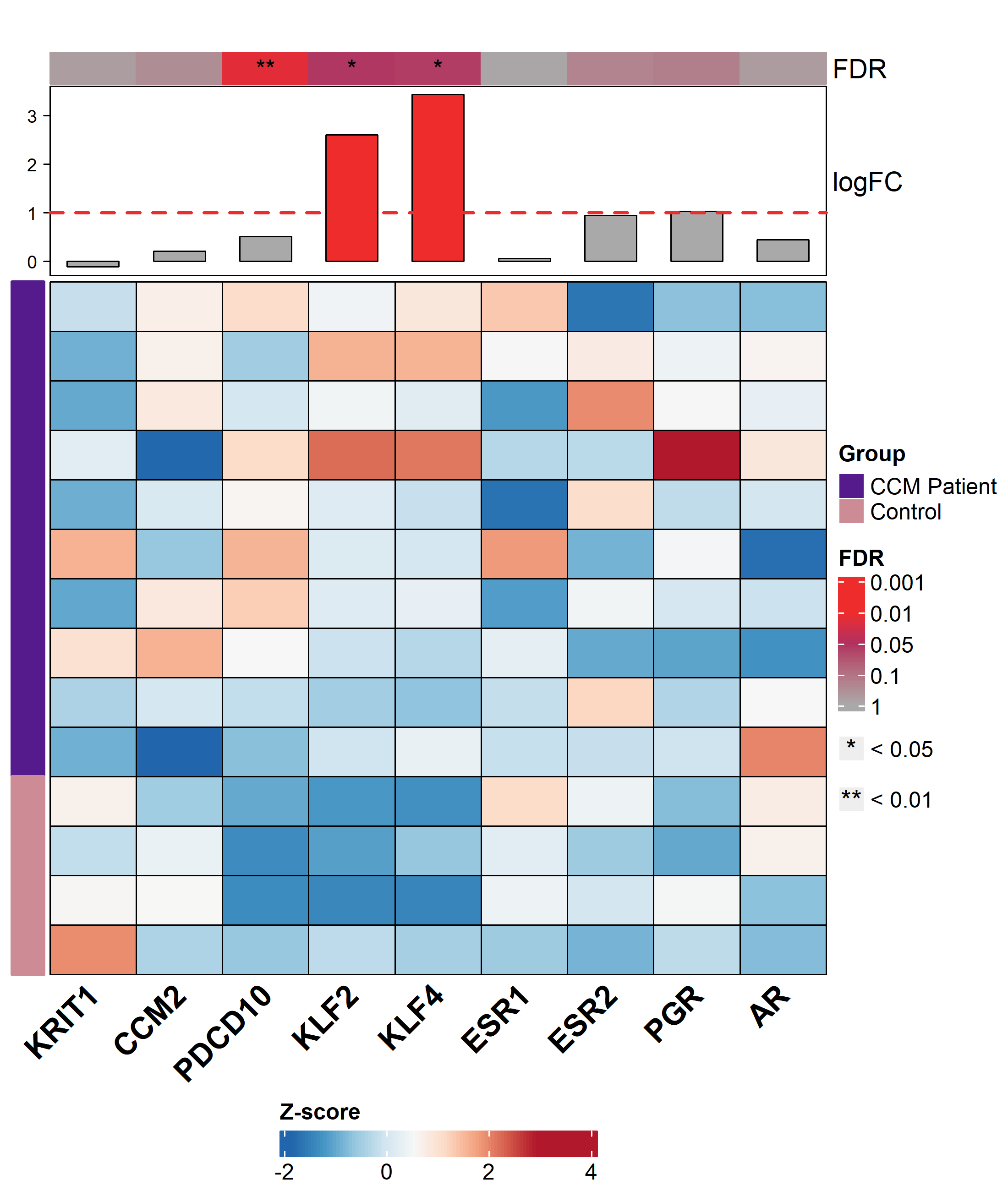
**

***Supplemental Figure III. Endothelial ESR1 deletion alone does not induce the development of CCM lesions in the brain.*** Macroscopic and micro-CT images show *Esr1^iECKO^* mice did not develop CCM lesions in the brain at postnatal day 12 (P12).

**
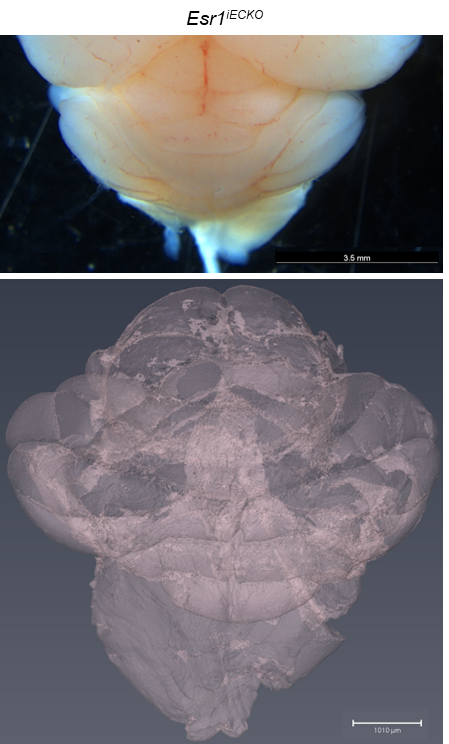
**

***Supplemental Figure IV.* Estradiol treatment of *Ccm1^iECKO^* mice at different doses. A)** Schematic of experimental design: Gene deletion in neonatal pups (postnatal day 1) was induced with 4-HT. Mice were treated with Estradiol (25 or 100 μg/kg) at P6 and brains were collected at P12 for analysis. **B-G)** Quantification of micro-CT analysis shows 25 μg/kg (B-D) and 100 μg/kg (E-G) of Estradiol treatment in *Ccm1^iECKO^* mice did not affect brain volumes**,** lesion volumes or numbers compared to sham-treated littermate controls. Error bars are shown as SD and significance was determined using t-test. ns not significant. *n*=4 per group.

***
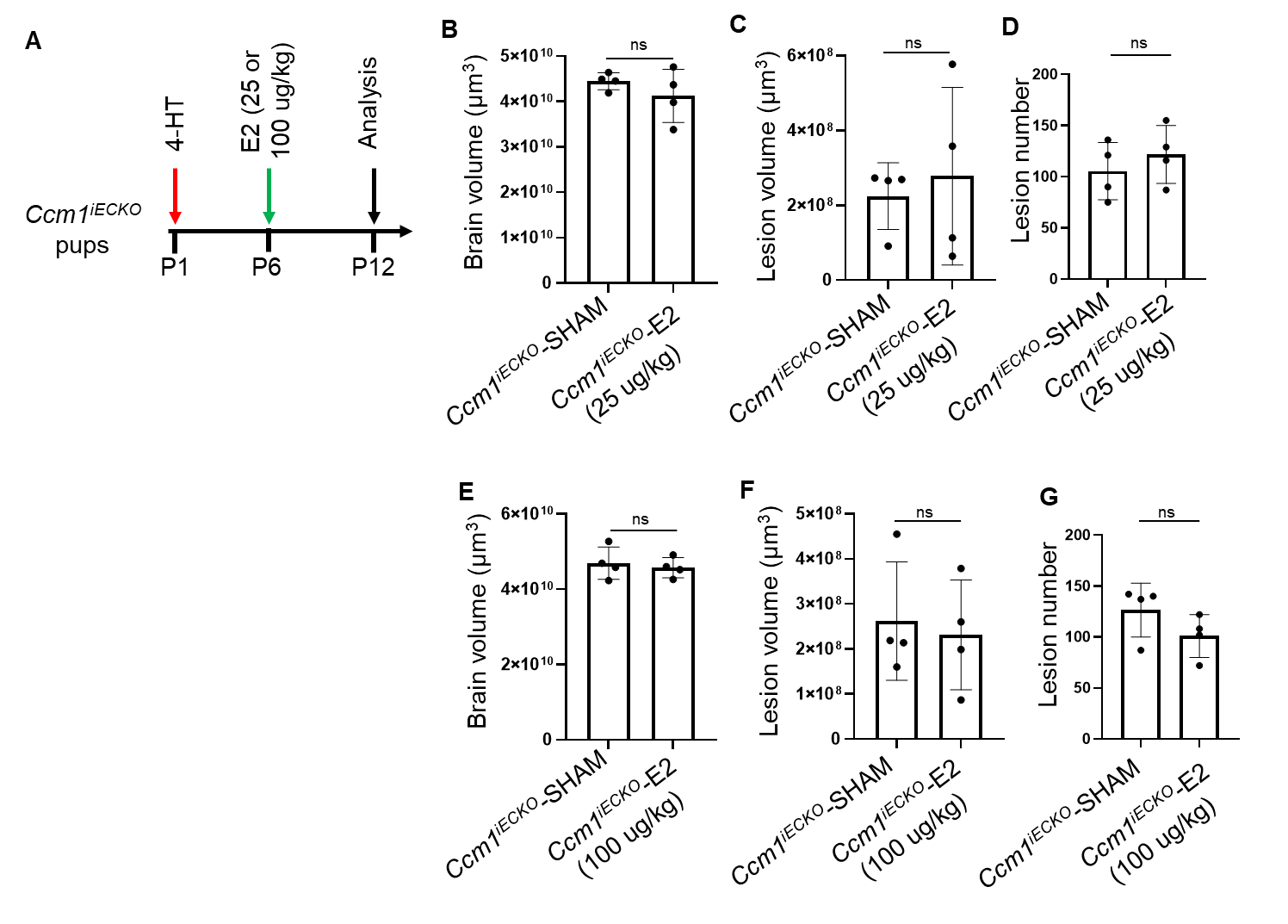
***

***Supplemental Figure V. CCM1 and ESR1 gene expression after its siRNA knockdown in HUVECs.*** *CCM1* and *ESR1* gene expression in siRNA-induced *CCM1* (si-*CCM1*) and *ESR1* (si-*ESR1*) gene knockdown in HUVECs. Error bars are shown as SD and statistical analyses were performed using one-way ANOVA. *p<0.05, **p<0.005, ***p<0.0001, ns not significant. *n*=3 each group.

**
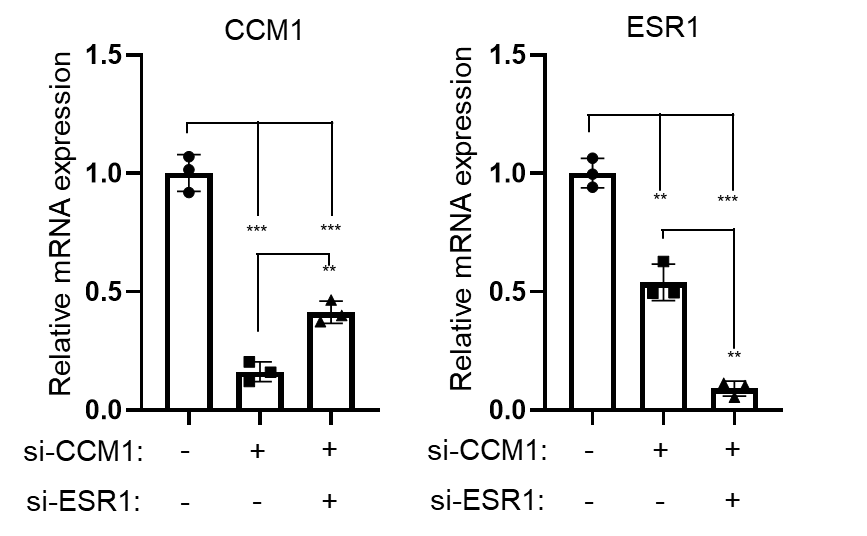
**
